# Plato's allegory of the cave and the paradigm of complete revascularization in STEMI

**DOI:** 10.1002/clc.23946

**Published:** 2022-12-20

**Authors:** Fernanda A. Justo, Bruna de Deus Herrera, Paulo R. Soares, Thiago L. Scudeler

**Affiliations:** ^1^ Instituto do Coração (InCor) Hospital das Clínicas da Faculdade de Medicina da Universidade de São Paulo São Paulo Brazil

To the Editor,

Primary percutaneous coronary intervention (PCI) is the standard therapy for patients with ST‐segment elevation myocardial infarction (STEMI). However, not all patients with STEMI have a one‐vessel coronary artery disease (CAD). Approximately half of the patients with STEMI have angiographically significant multivessel coronary artery disease (MVD).^1^

Observational studies discouraged complete revascularization during primary PCI in stable patients with STEMI and MVD.^2^ On the other hand, randomized clinical trials (RCTs) have shown improvements in composite outcomes of complete revascularization when compared to culprit‐lesion‐only PCI, mainly driven by nonfatal myocardial infarction (MI) and ischemia‐driven revascularization.^3^, ^4^, ^5^, ^6^, ^7^, ^8^, ^9^ However, these studies present several methodological limitations that restrict their external and internal validity.

First, what is the study hypothesis when we analyze the role of complete revascularization in patients with STEMI and MVD? Is the primary objective to evaluate the treatment of MI, coronary artery reperfusion, the evolution of MI, or the evolution of nonculprit lesion? In this scenario, the population studied is the one in the urgent care setting, but the trials have evaluated long‐term clinical events that are related to CAD itself, not to the treatment strategy during the STEMI event.

Second, the best way to assess the effect of an intervention is by choosing a single primary outcome. The composite primary outcome model is based on the premise that each component of the outcome is interchangeable, which rarely happens. None of the RCTs showed mortality reduction with complete revascularization, although none of the trials had enough statistical power to assess all‐cause or cardiovascular mortality. Moreover, ischemia‐driven revascularization or hospitalization for unstable angina is a faulty outcome for RCTs.^10^

Third, studies have indicated nonculprit lesions PCI with stenosis >50% or 70% by visual assessment or with hemodynamically significant stenosis as assessed by fractional flow reserve (FFR). However, arteries with less than 50% stenosis and therefore no indication for PCI are more likely to become unstable.^11^, ^12^, ^13^ Thus, cardiovascular events are more related to the degree of vulnerability of the coronary plaque than to the severity of coronary stenosis.^14^ It would be more appropriate to assess the degree of atherosclerotic plaque instability by optical coherence tomography or coronary intravascular ultrasound instead of assessing the hemodynamic severity of the plaque by visual assessment or FFR, as has been done in most RCTs. Moreover, diffuse coronary spasms are frequently present in the acute phase of STEMI, and this may lead to an overestimation of stenosis severity in nonculprit vessels.^15^

Fourth, natural‐history studies have shown that the risk of cardiovascular events in culprit and nonculprit lesions is not different. The Providing regional observations to study predictors of events in the coronary tree study showed that in patients with acute coronary events submitted to PCI, the risk of death from cardiac causes, MI, or rehospitalization due to unstable or progressive angina occurring during follow‐up was equally attributable to recurrence at the site of culprit lesions and to nonculprit lesions, primarily due to rehospitalizations for unstable or progressive angina.^11^ This data shows that complete revascularization might not be the preferred strategy in patients with STEMI and MVD. Moreover, most nonculprit lesions responsible for future events were angiographically mild at baseline (mean diameter stenosis, 32%).

Fifth, just as important as restoring epicardial infarct‐related artery patency is improving microvascular reperfusion, limiting the extent of irreversibly injured myocardium. Studies have shown that coronary microvascular obstruction in these patients is an independent predictor for cardiovascular events and adverse left ventricular remodeling after MI.^16^ Therefore, the focus of treatment should be primary PCI of the culprit vessel and optimization of cardioprotective therapy and not percutaneous treatment of epicardial coronary arteries with nonculprit lesions.

Sixth, many patients with STEMI are elderly and have a higher prevalence of renal failure, atrial fibrillation, peripheral arterial disease, left ventricular diastolic dysfunction, and arterial hypertension. Typically, this group of patients is not covered by the RCTs. Complete revascularization increases the radiation dose, contrast overload, and risk of contrast‐induced nephropathy. Therefore, complete revascularization increases the risk of adverse events in elderly patients with STEMI and MVD. In addition, patients older than 70 years of age have an increased risk for cardiogenic shock after STEMI, and manipulation of nonculprit lesions could pose an additional and questionable risk in this frail population.

Finally, although recent study‐level meta‐analyses have demonstrated the clinical efficacy of complete revascularization in patients with STEMI and MVD,^17^, ^18^ it is known that study‐level meta‐analyses have associated methodological challenges such as^19^: (1) they can lead to reporting statistically significant results despite the treatment's limited efficacy; (2) meta‐analytic confidence intervals are too narrow compared to the variability of treatment outcomes reported by individual studies; (3) literature‐based meta‐analyses are not a reliable measurement instrument; and (4) meta‐analysis averages out the differences among studies and leads to a loss of information. Therefore, study‐level analyses can lead to biased assessments.

Given the clear limitations of the studies involving stable patients with STEMI and MVD, the most recommended is still patient‐centered care, encompassing shared decision‐making with patients and clear scientific awareness between clinical and interventional cardiologists.

Knowledge as justified true belief must be based on solid evidence. This is well exemplified by Plato's myth of the cave. According to the philosopher, the process of obtaining knowledge encompasses two domains: the domain of sensible things and the domain of ideas. For Plato, reality is in the world of ideas—a real and true world—but most of mankind is in the world of sensible things, which are not perfect and, therefore, are not good enough to generate knowledge. Evidence‐based medicine reflects this dialectic. We are driven to create our absolute truths without stopping to question them. Transposing this reasoning to our critical analysis, we realize, therefore, that there is still a vacuum of information to establish a grounded knowledge regarding the benefit of complete revascularization in stable patients with STEMI and MVD. Remember that the information that an RCT provides the physician is based on statistics. RCTs reveal trends and average behaviors of a group. And, as we know, an individual's response often cannot be adequately predicted from the results of a group. Therefore, the application of any theory or generalized data to an individual patient fundamentally requires clinical judgment.
